# Recent Developments in Machine Learning for Mass Spectrometry

**DOI:** 10.1021/acsmeasuresciau.3c00060

**Published:** 2024-02-21

**Authors:** Armen G. Beck, Matthew Muhoberac, Caitlin E. Randolph, Connor H. Beveridge, Prageeth R. Wijewardhane, Hilkka I. Kenttämaa, Gaurav Chopra

**Affiliations:** † Department of Chemistry, Purdue University, 560 Oval Drive, West Lafayette, Indiana 47907, United States; ‡ Department of Computer Science (*by courtesy*), Purdue University, West Lafayette, Indiana 47907, United States; § Purdue Institute for Drug Discovery, Purdue Institute for Cancer Research, Regenstrief Center for Healthcare Engineering, Purdue Institute for Inflammation, Immunology and Infectious Disease, Purdue Institute for Integrative Neuroscience, West Lafayette, Indiana 47907 United States

**Keywords:** Deep Learning, Molecular Structure Prediction, Preprocessing Spectral Data, Peak Annotation, Mass Cytometry and Imaging, Omics, Clustering Methods, Transformer Networks, Gradient Boosting, Artificial Neural Networks

## Abstract

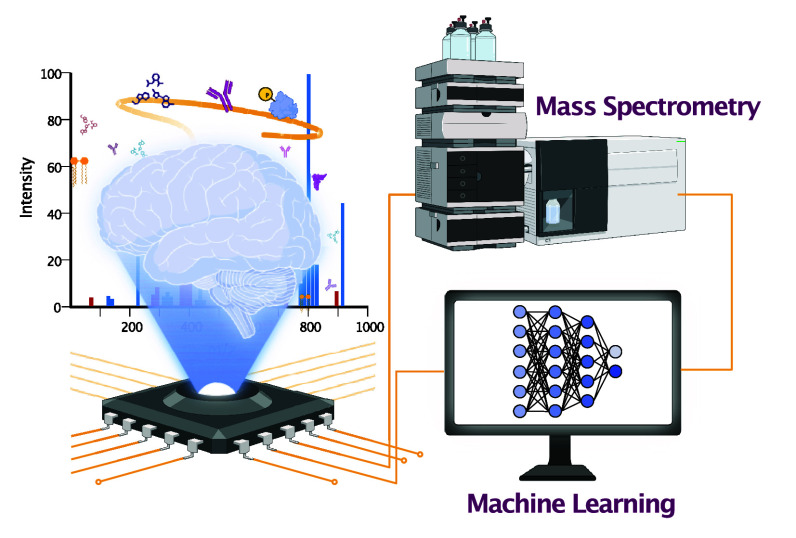

Statistical analysis and modeling of mass spectrometry (MS) data have a long and rich history with several modern MS-based applications using statistical and chemometric methods. Recently, machine learning (ML) has experienced a renaissance due to advents in computational hardware and the development of new algorithms for artificial neural networks (ANN) and deep learning architectures. Moreover, recent successes of new ANN and deep learning architectures in several areas of science, engineering, and society have further strengthened the ML field. Importantly, modern ML methods and architectures have enabled new approaches for tasks related to MS that are now widely adopted in several popular MS-based subdisciplines, such as mass spectrometry imaging and proteomics. Herein, we aim to provide an introductory summary of the practical aspects of ML methodology relevant to MS. Additionally, we seek to provide an up-to-date review of the most recent developments in ML integration with MS-based techniques while also providing critical insights into the future direction of the field.

## 1 Introduction

Mass spectrometry (MS) is a highly versatile and powerful analytical tool that can be used to detect, characterize, and quantify a wide range of analytes based on their observed mass to charge ratio (*m*/*z*). MS techniques and the associated hardware have been developed for a variety of applications, covering almost all areas of science, ranging from archeology to drug discovery to forensics. Computational advancements for MS data analysis have dramatically evolved over the years, as a plethora of chemometric and cheminformatic tools that have been developed for MS data interpretation. Routine data analysis operations like peak picking and identification are indispensable facets to modern MS-based analyses.

However, despite recent advancements in computationally mediated MS-based techniques, significant challenges in MS data analysis still exist. For example, many MS-based approaches still rely on the utilization of reference libraries for analyte identification.^1^ Such libraries are constructed with authentic reference standards and their corresponding tandem-MS (MS/MS) spectra, depicting the observed fragmentation pattern of the analyte precursor ion to collision induced dissociation (CID). Unfortunately, analyte identification via library matching has inherent limitations. One such limitation includes reliably capturing the large chemical space of unknown analytes given the nonlinear relationship between the precursor ion structure and associated product ions generated via tandem-MS. This challenge is further compounded by the demand for increasingly long lists of target analytes to be confidently identified in samples containing complex mixtures where traditional computing methods are inefficient to capture the diversity of MS data.

**Figure 1:**
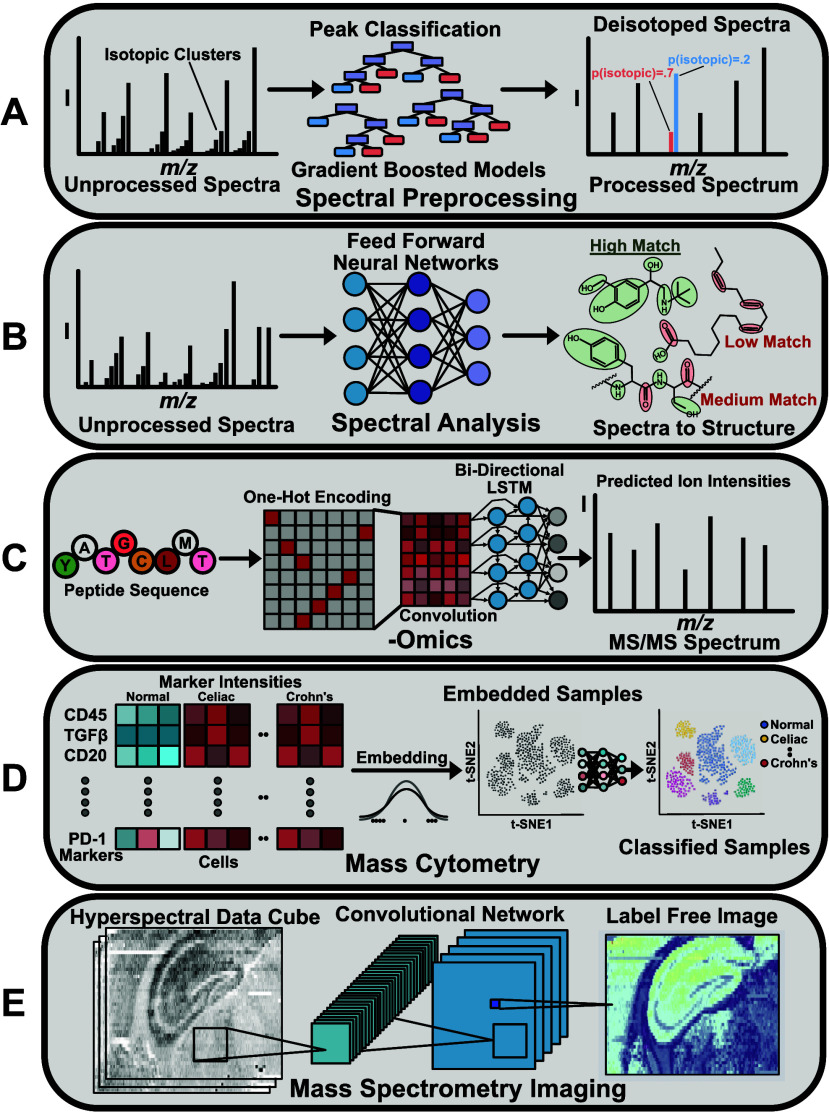
Overview of major uses of machine learning for applications in mass spectrometry and representative approaches. (A) Preprocessing of mass spectra by using gradient boosted decision trees for classifying peaks in isotopic clusters. (B) Spectral analysis using a feed forward neural network to predict structural features for analytes from their mass spectra. (C) Prediction of peptide product ion abundances using a deep learning pipeline analogous to DeepSCP. (D) Embedding and classification of tissue samples analyzed using mass cytometry. (E) Mass spectrometry imaging mediated using a convolutional neural network.

To address these shortcomings, machine learning (ML) methods have been adopted to both interpret and predict patterns in the MS data. ML methods belong to the general class of data-driven problem solving methodologies. There are no rules specified to relate input to output; ML methods compute sets of numerical weights and rules to relate input to output that are iteratively modified based on the training data. Several ML methods have been successfully implemented in chemical sciences, such as chemical reactions,^2^ quantum chemistry,^3^ and drug discovery.^4,5^ Additionally, new deep learning architectures have been implemented throughout analytical chemistry.^6^ Due to recent implementations and advancements of ML within the MS community, this review aims to serve nonexperts in either ML or MS techniques to understand synergistic opportunities of combining ML and MS techniques (Figure 1). Specifically, we provide a detailed outline of fundamental ML concepts and methods along with their historical and recent applications in MS studies. To conclude, we offer critical insights regarding potential advancements associated with ML and MS combined methodologies along with future directions for this new area in measurement science.

## 2 Machine Learning Models and Terminology

We begin by briefly introducing the most popular ML models and artificial neural networks (ANNs). In general, this section serves as a concise tutorial, educating readers on ML terminology along with the introductory concepts of ML methods. With the basic knowledge provided here, readers should be able to select appropriate ML models for specific MS applications detailed in Section 3.

### 2.1 Preprocessing and Featurization

Before utilizing ML methods, it is commonplace to incorporate additional techniques such as preprocessing and featurization to annotate MS-based data. Preprocessing refines the data to make them more suitable for ML models. Common methods of preprocessing training/validation/test data include normalization, scaling, and encoding to reduce bias, such as those introduced by variations in instrumentation and inter/intraday experimental variation. In the context of MS, normalization is often accomplished by employing isotopically labeled internal standards. On the other hand, featurization can be employed to transform MS-based data into new variables or formats that best represent the original spectral data. In the context of MS, featurization can be accomplished by binning of *m*/*z* abundance values, vectorizing analyte topology via molecular fingerprinting strategies, representing proteins as a sequence of one letter amino acid abbreviations, or utilizing graph data structure to represent compounds. Depending on the choice of ML method, featurization can be an essential step, as not all datatypes and ML models are initially compatible with each other. Additionally, featurization techniques such as molecular fingerprints can be highly sensitive to chosen parameters such as bit radius, vector length, and the underlying nature of the algorithm itself.^7^ Therefore, transforming data into a suitable format via featurization ensures that the ML model can learn from it effectively.

### 2.2 Supervised and Unsupervised Machine Learning

In general, there are several subcategories of the ML model training methodology. Supervised and unsupervised learning methods represent the most common dichotomy. In brief, supervised ML methods are trained using labeled data, meaning that each input data point is paired with a correct output. Supervised ML models learn to predict outcomes based on these data. In the context of MS, examples of input and output labels can be peak heights, sample identity, peak classification (i.e., isotopic vs monoisotopic peak), etc. Conversely, unsupervised learning operates on unlabeled data, often inferring patterns. Unsupervised learning methods are most commonly used for tasks such as clustering and dimensionality reduction regarding MS applications and are a good way to generate labeled training data for specific supervised training tasks.

### 2.3 Ensemble Learning

**Figure 2:**
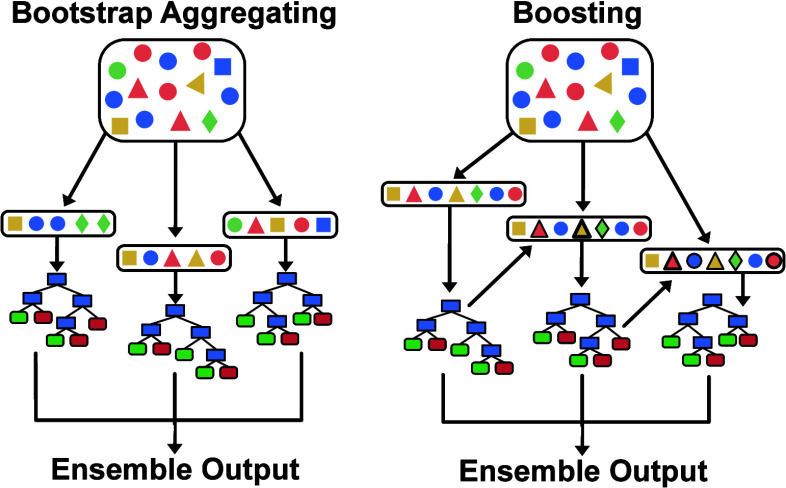
Schematics for ensemble learning methods. On the left, bootstrap aggregating is shown, where data permutations are used to train machine learning models in parallel. On the right, bootstrap aggregation (boosting) is shown, where machine learning models are trained sequentially on the initial (same) training set. The performance of each model affects the weighting of the training set used (shown by bold shapes) during the training of the subsequent model. The output is a combined result from all models.

Ensemble learning combines multiple ML models to work together, effectively operating as a single unified model. Bootstrap aggregating, also known as bagging, and boosting are frequently used ensemble learning (Figure 2). Bootstrap aggregating has multiple variations but, in general, involves the use of data permutations for training a large number of models. Data permutations are performed in bagging with replacement. In other words, training sets may consequently contain repeats of the same data point. These models are trained in parallel, and then, predictions are made by taking averaged outputs as the ensemble model. Boosting involves sequential model training with weighted sums of the models being used for the resulting ensemble model. Boosting variants are often defined in the manner in which the weighting term and error for individual models are calculated. Additionally, it is often the case that data points for new models are weighted based on their contribution to errors on prior models. One popular type of method for boosting in MS-based applications is called gradient boosting, wherein (pseudo)residuals of error, the training data incorrectly predicted, are used to iteratively train the weak models that will comprise the ensemble model.

### 2.4 Linear, Logistic, and Other Simple Regression Models

Linear and logistic regression models are a class of models that learn a simple linear or logistic function to predict the value of an output variable (*y*) given an input variable (*x*). In linear regression, the function that relates input and output data is linear (e.g., in the form *y* = *mx* + *b*), and the algorithm tunes the slope (*m*) and intercept (*b*) based on the provided training data points. The output line of a linear regression model is often termed the line of best fit for that specific collection of data. To evaluate the accuracy of the linear model, a cost function is used. The cost function is a summed distance metric between all predicted points’ *y*-values (on the line equation) and all actual points’ *y*-values (in the data set). The smaller the value of the cost function, the closer the linear model’s predictions are to the actual data points. A common cost function used to evaluate linear regression models is the mean squared error (MSE). In a similar manner, logistic regression models attempt to find a logistic function (e.g., ) which best fits the training data. A commonly used cost function for logistic regression is the log loss. In addition to linear and logistic regression models, there are other categories of simple regression models that attempt to optimize functions for individual data sets. Examples include polynomial regression models that find n-degree polynomial functions to model data and exponential regression models that find functions to model data sets that exhibit exponential growth. Based on collected data sets, users must choose between functional regression model types, selecting the best possible model type to model that data form. Though linear models can be considered simple ML models, their use for MS applications continues to be prevalent as these models are highly interpretable.

### 2.5 Support Vector Machine and Variants

Support vector machines (SVM) are a category of ML models which attempt to find decision boundaries (i.e., a line or function) that separate data points based on their labels. Representing a unique aspect of SVM models, decision boundaries are not simple one-dimensional boundaries. Instead, SVM decision boundaries are hyperplanes that attempt to maximize the separation distance between class labels. To accomplish this task, decision boundaries are first fit to the data points of different classes, starting with the data points closest to the decision boundary. These data points on the hyperplane boundaries are known as the support vectors. In general, support vectors help determine the degree of separation provided by the hyperplane. Importantly, SVM models have a statistical ability to assess outliers of certain label classes that may cross over into the hyperplane region, representing a significant advantage of SVM models. While SVM models have hyperplane decision boundaries that are linear in nature, SVM models have been recently adapted to create nonlinear decision boundaries given a complex relationship among the data using what is known as the kernel trick. In brief, the kernel trick employs a similarity function to transform the data to a higher dimensional representation, where hyperplanes can be found to separate data based on label classes. Additionally, SVM has been extended to regression tasks and is typically refered to as suport vector regression.

### 2.6 Tree-Based Models

Tree-based models refer to ML models that use a tree-based data structure as a guide for decision-making. The two prominent subcategories of tree-based models include decision trees and random forests. To construct decision trees for a given training data set, specific values of particular features are repeatedly divided to maximize information gain. In a general binary classification example (labels A and B), each division should reduce the amount of data set classification uncertainty by splitting data such that label assignments are made to as much of label A and label B as possible. To make a prediction using a decision tree, an individual data point is traced through the tree, following the pathway determined by its features, until it reaches a leaf node for a prediction (decision). Due to the inability of the model to correctly generalize data not included in the known training data set, overfitting is especially common in taller (or deeper) decision trees. In other words, overfitting is an undesirable ML behavior in which only known training data can be accurately predicted. To combat overfitting, a technique called pruning is often used to reduce the length of extraneous branches to promote better generalization; this is also a form of hyperparameter tuning of decision trees. Random forests are ensembles of decision trees that have been trained using a version of bootstrap aggregation (bagging) described earlier in the manuscript.

Due to their prevalence, it is also worth describing some ensemble methods that utilize decision trees by boosting. The eXtreme Gradient Boosting (XGBoost) library developed around a decision tree based boosting algorithm that, unlike “vanilla” gradient boosting, utilizes a second order Taylor approximation of the loss gradient for weighting the splitting decisions when building trees with the addition of regularization that penalizes model complexity to avoid overfitting. Catboost is another tree boosting library that is designed to effectively handle categorical variables with built in regularization. These regularizations include symmetric tree structures that utilize the same feature-split at each node at the same level to aid with generalizability. Catboost also incorporates various ways to utilize a natural ordering to categorical features. Lastly, a light gradient boosting machine (LightGBM) differs from other methods as it sequentially divides at individual nodes rather than uniform layers. In turn, LightGBM combines correlated categorical features to reduce dimensionality and triages training data by selecting points contributing larger loss gradients via a technique called exclusive feature bundling and gradient-based one-side sampling, respectively.

### 2.7 Embedding Methods

When working with data in higher dimensions, it is often preferable to reduce dimensionality. Importantly, data dimensionality reduction can aid in data visualization or feature selection. To accomplish this, a variety of ML embedding methods have been developed. Such methods include matrix factorization methods like principal component analysis (PCA), t-distributed stochastic neighbor embedding (t-SNE) and uniform manifold approximation and projection (UMAP). Moreover, dimensionality reduction techniques are often used in tandem with clustering algorithms to assign classes to data points. Dimensionality reduction prior to clustering can be essential, as distance metrics tend to lose efficacy in higher dimensions. It is important to note that dimensionality reduction methods can not only remove dimensions but also create new dimensions that are combinations of previous dimensions.

Matrix factorization methods can identify factors that allow for linear mapping between matrices of differing dimensionality. The most common method of matrix factorization used for MS-based applications is the PCA, though other methods, such as singular value decomposition and non-negative matrix factorization, have been used to a great degree as well. PCA functions by mapping data to a new coordinate system based on variation in the data, with the dimensionality of the new coordinate system being defined by the individual principal components. The goal of PCA is to find a coordinate system that explains as much variation in the data as possible by using a low number of dimensions that are linear combinations of the input dimensions. Two other common embedding techniques are t-SNE and UMAP. Unlike PCA, t-SNE and UMAP utilize nonlinear transformations to achieve embedding and preserve local topology, with UMAP preserving global topology in addition.

While UMAP has been replacing the use of t-SNE as of recently, PCA can still be beneficial to use, although careful consideration is needed when choosing the appropriate embedding method. Specifically, one may find featurization based on variation useful while at other times, *m*/*z* peaks with smaller magnitude may contribute little to overall variance but could be of great significance. A thorough review regarding the applications of these methods in the context of mass spectrometry imaging (MSI) has been published elsewhere, discussing the relevant methods and their usages in detail.^8^ We note that neural networks, such as autoencoders, can also be used for embedding, and these strategies are discussed in Section 2.9.3.

### 2.8 Clustering Algorithms

Typically categorized as unsupervised methods, clustering algorithms aim to both cluster and label data points. Typically, the clustering of MS data is performed after preprocessing by using an embedding technique such as PCA or UMAP. Importantly, clustering can be used for not only differentiating samples but also predicting a specific class for a new sample. Common clustering algorithms include k-means clustering, hierarchical clustering, Gaussian mixture models (GMMs), k-nearest neighbors (KNN), and density-based spatial clustering of applications with noise (DBSCAN). Both k-means and GMMs cluster the data to a set number of clusters based on the distance of data points from randomly initiated cluster centroids, whereas hierarchical clustering splits or groups data based on similarity. While these previous methods are unsupervised, KNN is a supervised method that assigns unlabeled data points to clusters based on their distance from labeled data. DBSCAN is also an unsupervised method that, unlike density-based clustering methods, k-means, and GMMs, does not require the number of clusters to be defined *a priori*. Additionally, DBSCAN does not use unbounded distance-based metrics to assign data to clusters, allowing for outliers or noise to remain unassigned, unlike in k-means and GMMs.

### 2.9 Artificial Neural Networks and Deep Learning

**Figure 3:**
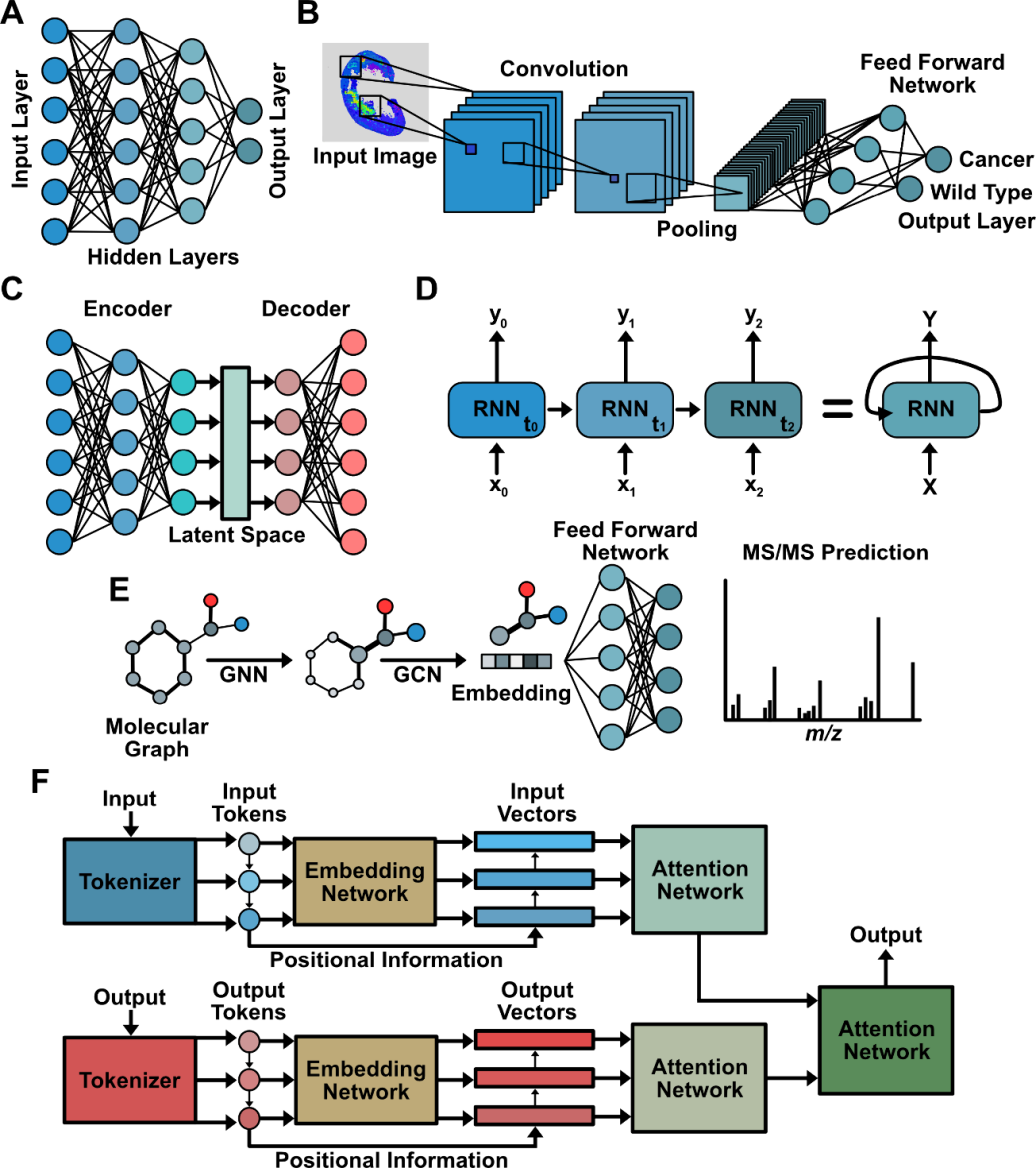
Schematics for artificial neural networks used for mass spectrometry. (A) Generalized depiction of a multilayer perceptron. (B) Depiction of a convolutional neural network classifying a mass spectrometry imaging sample. (C) Generalized depiction of an autoencoder network, displaying the process of encoding into and decoding from a latent space. (D) Generalized depiction of an unrolled and rolled recurrent neural network. (E) MS/MS spectral prediction of a generalized molecule by using graph neutral networks for molecular graph processing, graph convolution networks for molecular graph embedding, and a feed forward network for final spectral prediction. (F) Generalized depiction of a transformer network with the attention mechanism abstracted.

Artificial neural networks (ANNs) (Figure 3) are a class of ML models that attempt to emulate biological neurons known to be the basic decision-making units in humans. Neural networks have a fundamental node unit composed of three main parts: (1) a numerical scalar (weight), (2) an activation function, and (3) a set of input connections from and output connections to other nodes. Briefly, the node receives numerical input from its input nodes, scales this input by its weight, determines the output by passing the scaled input into the activation function, and then passes the activation function output to its output nodes.

In general, nodes are grouped together into layers. Layers and their connected architectures are established based on network location and collective node function. Specifically, the layer located at the start of the network is called the input layer, while layers in the middle and end of the network are referred to as hidden and output layers, respectively. Additionally, ANNs can be used as either classification or regression models, depending on the activation function used for nodes in the output layer.

To better understand how ANNs function from a mathematical perspective, we present the following example of a feed-forward network trained via backpropagation. During network training, data points are split into batches and then traverse through the network from input layer to output layer, resulting in mathematical weights being applied to each data point at each layer until they reach the output layer, resulting in a prediction. After the prediction is made, a loss function is used to calculate the variation of the prediction from the ground truth value. Once all data points in a batch have their predicted loss values calculated, the model’s weights are updated starting at the last layer by calculating the gradient of the error to each weight in a layer and then adjusting the weight in the opposite direction of the gradient scaled by the learning rate. This process is repeated for each layer, back to front, where the gradient for each latter layer is used to calculate the gradient for each former layer. This results in the entire model’s weights being updated in a direction which should minimize error for the provided data. By changing the batch size and learning rate, the scope and degree of weight updates can be controlled.

Deep learning refers to networks containing multiple hidden layers that are fixed between the input and output algorithms and are responsible for nonlinear transformations of relating input to output data to accomplish the desired tasks. Specific examples of ANN layers include convolution and recurrent layers as part of convolution and recurrent neural networks, respectively. Notably, the sizes and connectivity of each layer are optimized during training, which is also referred to as hyperparameter tuning. In short, neural network training essentially incorporates determining the weights that relate input(s) to output(s) in training data sets. We note that a recent review provides an in-depth discussion regarding the usage of ANNs on deep learning and its applications to analytical chemistry.^6^ As discussed in additional detail below, we provide details of common neural network architectures used for MS related tasks.

#### 2.9.1 Multilayer Perceptron

Multilayer perceptrons (MLPs) (Figure 3a) are some of the simplest types of ANNs. MLP composition is defined by an input layer, hidden layers, and an output layer. MLPs belong to a class of ANNs known as feed forward networks. Feed forward networks entail exclusively forward progression of information from the input layer to the output layer without cycling back to any previous layers. MLP behavior modification can be achieved via various existing activation functions for neurons.

#### 2.9.2 Convolutional Neural Networks

First popularized by the computer vision field, convolutional neural networks (CNNs) (Figure 3b) have since become widespread.^9^ Most often designed as feed-forward networks, CNNs can be generally described as a subset of MLPs. However, the defining feature of CNNs is the use of convolutional and pooling layers. Convolutional layers utilize a matrix with learned weightings called a kernel. In brief, the kernel produces a feature map via dot products with the input. Notably, the kernel is smaller in dimension than the input matrix and operates at all positions across the input matrix, capturing overlapping submatrices of the kernel size. Due to the nature of the matrix operations, the dimensions of the feature map will be smaller than that of the input. Consequently, many convolution layers apply a technique known as zero padding to preserve dimensionality. Typically, after a given number of convolution layers, a pooling layer is used to reduce the dimensionality of the information that is being transferred. Pooling utilizes a similar approach to kernel operations, where a grid is passed over the input data. However, the operation for pooling does not contain learned parameters but rather transfers either the maximum or average value in the grid to the next layer. The grid dependent behavior of CNNs that makes them well suited for computer vision tasks has translated to their prevalence in MSI, although 1-D convolutional approaches and other applications outside MSI exist within the context of MS.

#### 2.9.3 Autoencoders

Autoencoders are another type of ANN architecture that frequently operates as feed-forward networks (Figure 3c). Autoencoders are trained in an unsupervised manner, where the task of the network is to reduce the dimensionality of input data via “encoding” and subsequently recover the original input through “decoding”. The input data is encoded into a lower dimensional representation of the relationship between input and output of the neural network known as latent space. Once trained, latent space variables can be used for tasks such as clustering to reduce dimensionality much like methods relying on PCA or t-SNE. Alternatively, it is common for trained decoders to be used in a generative manner, such that a combination of latent variables yields a desired output, such as a structure of a molecule. Various forms of autoencoders exist and are typically defined by how they are trained and the regularization of their latent space. One commonly used form of autoencoders are variational autoencoders. In short, variational autoencoders introduce variation into the encoding and decoding process. This is done by utilizing an additional regularized layer with learned mean and standard deviation components for sampling the latent space.

#### 2.9.4 Recurrent Neural Networks

Unlike feed-forward networks, recurrent neural networks (RNNs) (Figure 3d) form cyclical connections between nodes. ANNs that belong to the class of RNNs are characterized by their ability to leverage data in a sequential manner, with previous inputs providing context for future inputs in the sequence. Many variants of RNNs exist, such as long short-term memory (LSTM) networks and gated recurrent unit (GRU) networks, and their architectures are typically defined using gated units. Gated units function by regulating the flow of information throughout the network, and can serve in various manners, such as limiting information passed into units or out of a cell. In the case of LSTMs, each cell contains an input gate, a forget gate, and output gate. Each gate has learnable weights and is responsible for scaling the previous hidden state output, combining, and modifying the scaled hidden state and current input to produce the memory cell state, and utilizing the previous hidden state, memory cell state, and input to yield the output vector for the input, forget, and output gates, respectively. RNNs have become more popular for MS tasks in recent years and have been employed for MSI tasks,^10^ decoding amino acids from MS spectra,^11^ and MS/MS spectra prediction.^12^

#### 2.9.5 Graph Based Neural Networks

In computer science, graphs are defined data representation architectures with edges and nodes. Although sparsely used in the context of MS, much work has been done with chemical applications using graphs. Molecular graphs are a common graph-based representation used for chemistry, where the edges represent bonds, and nodes represent atoms. Beyond identifiers, nodes and edges can contain other forms of numeric information such as distances between atoms being stored in edges. To handle and leverage inputs in the form of graphs, many graph-based networks (Figure 3e) have been developed. Graph neural networks (GNNs) operate on graphs and modify their representations based on predictions. In turn, GNNs will often have their property embeddings pooled and summed for predictions performed by other ANN layers. Various other types of graph-based neural networks have been developed, with a notable example being graph convolutional networks (GCNs). Like CNNs, GCNs are networks containing graph-convolution layers and operate in an analogous manner. Graph-convolution layers can be used to reduce the number of elements in a graph, much like how convolutional layers would if zero padding is not used. Graph based neural networks are a powerful tool when dealing with structural information, with the most notable example in chemistry being AlphaFold.^13^

#### 2.9.6 Transformer Networks

A class of neural networks that further builds upon the use of positional context leveraged by RNNs is transformer networks (Figure 3f). However, unlike RNNs, transformers use a mechanism called attention to process input either as a sequence or altogether while learning parts of input that are most important based on training, i.e., where attention should be given. The architecture for transformers is rather complex and comprised of many components. While specific details regarding the architecture of transformers are out of the scope for this review, the overall design of transformer networks can be described as stacked encoder-decoder networks. However, transformers have started becoming more prevalent in the proteomics literature, replacing the use of RNNs, and we would like to encourage others to consider applying transformers in cases where RNNs have proven successful.

## 3 Machine Learning Applications for Mass Spectrometry

To address the challenges associated with MS data interpretation, ML methods have been developed and employed for decades. Highlighting their versatility and utility, ML methods can be applicable to an extensive range of MS-based approaches and platforms. In this section, we discuss recent developments in ML that have been applied to several MS-related studies.

### 3.1 Spectral (Pre)processing

Prior to MS data interpretation with ML methods, proper data preprocessing is essential. Preprocessing involves the initial conversion of raw instrument data to more interpretable formats. To date, several preprocessing techniques with chemometric origins have been established. However, deep learning applications have been somewhat limited in mass spectrometric approaches as deep learning strategies often circumvent the need for spectral preprocessing.^14,15^ Nevertheless, multiple ML preprocessing methodologies have been reported outside the context of feature reduction. While software such as XCMS is capable of complex tasks such as retention time (RT) correction and peak detection by using algorithmic approaches coded by experts, ML offers the potential to provide greater fidelity by leveraging nonlinear relationships extracted from the data. However, ML models may require differing amounts of training data to overcome the performance of these traditional approaches. We discuss various nonlinear ML approaches to preprocessing using decision-tree ensembles, SVM, and neural networks.

Deisotoping is a common feature reduction strategy used across many MS-based fields, including proteomics. In short, deisotoping involves the removal of noninformative mass spectral peaks that are due to naturally occurring uncommon heavy isotopes. Importantly, deisotopes improve spectral annotation and peak identification methods by reducing spectral complexity. Utilizing tree ensemble models, in addition to other ML mediated features, the python package MEDUSA has the preprocessing feature of deisotoping high resolution mass spectrometry (HRMS) data.^16^ Deisotoping with MEDUSA is performed using either random forest, eXtreme Gradient Boosting (XGBoost), or CatBoost and it functions as a binary classifier to predict whether a peak pair can be attributed to isotopes or not. A package written in R, called AutomRm, allows users to train and use ML models for the preprocessing of data obtained using multiple reaction monitoring (MRM) in conjunction with liquid chromatography (LC).^17^ AutomRm was reported to have a comparable performance for peak selection and reporting when using a single hidden layer MLP, random forest, SVM (polynomial), or XGBoost.^17^ Although all models were comparable, the random forest displayed a superior combination of classification fidelity and speed. While using random forest as the underlying ML model, AutomRm was benchmarked against various software with MRMPROBS^18^ performing comparably in terms of the standard deviation of reported peak areas for standard metabolite replicates, albeit a slightly lower standard deviation when using AutomRm. However, regarding the accuracy and specificity of peaks being reported, MRMPROBS suffers from a marked decrease in performance when compared to AutomRm, likely due to the limited use of only five extracted peak features and scores for the underlying multivariate logistic regression model.

Mass spectral denoising is an additional form of spectral preprocessing that has gained considerable attention in recent years. For example, conditional generative adversarial networks have been reported for mass spectral denoising and demonstrated successful removal of low-mass noise peaks from the mass spectra generated for a series of polymers.^19^ In this study, polyethylene glycol (PEG) polymers with varying molecular weights were first analyzed using matrix-assisted laser desorption/ionization time-of-flight (MALDI-TOF) MS. Next, denoised pseudomass spectra containing only PEG peaks as ground truths were utilized for training the network. Noise peaks were then added to the pseudomass spectra and used as inputs to a generative network. This augmented data set was subsequently trained to generate denoised pseudomass spectra, while a discriminator network was trained to determine if the generated spectra were authentic by using ground truth spectra. Demonstrating method validity and versatility, the effects of the MALDI matrix and polymer composition were tested. Specifically, the network was tested for performance on both PEG and polyethylene terephthalate (PET), with the PET mass spectra denoised both before and after degradation via UV irradiation. Additionally, two different MALDI matrices were used for PEG and PET ionization. Together, these results illustrate method robustness, as it can effectively perform on both a polymer and matrix not used during training.

### 3.2 Spectra Analysis for Compound Identification

Many routine MS-based identification tasks involve peak annotation, determination of the elemental compositions, and subsequent extraction of structural information from product ions observed in tandem mass spectra. Peak annotation can be applied to peaks observed in MS or MS/MS spectra. Additionally, software for calculating elemental composition of compounds based on accurate mass measurements is widely available. Various spectral databases have been developed to identify known compounds, such as metabolites, based on mass spectral data.^20^

However, such databases suffer from inherent limitations regarding the structural identification of unknown analytes. From these databases, experimentally generated and reported library mass spectra can either be compared directly based on observed ion *m*/*z* and/or compared considering additional information, such as RT for LC-based experiments and observed fragmentation patterns of product ions generated from CID of analyte precursor ions. Though many compounds can be identified through database searches, automated spectral matching can fail due to an absence of reference data, lack of structural information in the reference data (i.e., such as the lack of molecular weight information), or complications like noise, resolution, or mismatched experimental conditions. On the other hand, CID spectra can be predicted using rule-based software, serving as a potential approach to supplement mass spectral databases.

Below, we discuss how ML has impacted peak annotation, determination of compound elemental compositions, structural prediction, and mass spectral prediction. Though outside the scope of this review, recent reviews have been published detailing compound identification,^21^ computational resources for metabolomics,^22^ and deep learning for MS/MS based structural prediction.^23^ Additionally, omics related research related to structural prediction is also detailed in Section 3.5.

For proper analyte classification, first, the elemental composition must be accurately determined. In a recent report, a tree boosting ML model with a Light Gradient Boosting Machine was used to label peaks in high-resolution TOF mass spectra of inorganic complexes by peak pattern identification combined with elemental and nonmetal molecular database.^24^ Importantly, the model was validated on TOF data generated using atom probe tomography (APT) and secondary ion mass spectrometry (SIMS) following computationally generated data training. In addition to ML mediated deisotoping, the MEDUSA software package also contains deep learning models for generating molecular formulas from HRMS spectra.^16^ Various models were trained on computationally generated data with the best performance using recurrent neural networks, specifically two bidirectional LSTM models. The first LSTM model is responsible for classifying the elements, while the second serves as a regression model to predict the number of each element. The authors of MEDUSA highlight the novelty of their approach as the first fully automated mass spectrometry analysis platform without strict constraints on the number of observed elements for a mass range below 2000 Da.

Conversely, rather than predicting molecular formulas with ML, logistic-regression on proposed molecular formulas from HRMS and MS peak related features was used to classify proposed formulas as true or false.^25^ In an additional example, MS and Fourier transform infrared (FTIR) spectra were utilized in conjunction with a ML model for the prediction of functional groups in analytes.^26^ This was achieved by training an autoencoder on concatenated MS and FTIR spectra, and using trained latent vectors from the autoencoder as inputs for an MLP used to predict and experimentally validate functional groups in a multiclass multilabel manner.^26^ Another method, MassGenie, has also been developed for small molecule structure prediction based on the input mass spectra. This model works by using a transformer network and a variational autoencoder for generating potential candidates.^27^ While this work did not perform especially well on experimental mass spectra due to noise not used in the training data, the underlying principles provide a strong starting point for future work. In a final example, we note that SIRIUS 4, a software suite designed for identifying metabolites, has been trained on tandem mass spectra obtained in positive (protonated or metal-adducted analytes analyte ions) and negative (deprotonated or anion-adductedd ion types) ion modes by using SVM and deep neural networks.^28^

Due to incomplete databases, the ability to accurately identify the structure of a compound based on mass spectra is challenging. To combat this challenge, augmenting existing databases with computationally generated mass spectra could provide a potential solution, a task that ML is suitable to handle. In particular, multiple ML methods have been developed for the prediction of small molecule and protein mass spectra. Methods for mass spectra prediction using ML include the prediction of electron impact mass spectra (EI-MS) by using MLPs^29,30^ and GNNs,^31^ MS/MS spectra of ionized small molecules using molecular fingerprints with random forest approach,^32^ of ionized metabolites by using a GNN,^33^ and CID and higher-energy C-trap dissociation (HCD) MS/MS spectra of ionized proteins by using an MLP,^12^ a bidirectional RNN,^12^ a transformer network,^34^ or a pipeline using a bidirectional LSTM and transformer for phosphoprotein fragment ion abundance prediction.^35^ Although architectures such as RASSP^29,3^ (GNN) outperform more basic ones such as NEIMS^31^ (MLP), it is important to consider and investigate data scalability if retraining is needed for repurposing existing models. Additionally large transformer networks such as Prosit Transformer^34^ require large amounts of training data to achieve their reported performances, without the finetuning of pretrained models. For CID and HCD MS/MS spectral prediction, Tiwary et al. developed an MLP for protonated protein analysis. Interestingly, Tiwary et al. found that the reported MLP was less computationally expensive compared to previously utilized approaches like RNN, and that MLP could also be used to process proteins of varying length using an iterative sliding-window approach.^12^

In a final example, a bootstrapped decision tree model has been reported for predicting the formation of diagnostic product ions upon gas-phase ion–molecule reactions.^36^ This model was trained in a manner such that given the molecular fingerprint of an analyte, the model determines the outcome of ion–molecule reactions occurring within the collision cell of a mass spectrometer. For example, the model accurately predicts and interprets how the trapped analyte ions react with 2-methoxypropene, yields either diagnostic product ions or uninformative proton transfer product ions.^36^

### 3.3 Sample Classification and Clinical Diagnostics

The use of ML to classify clinical patient samples based on MS data is an essential task for several applications. Fortunately, this approach is generalizable and has been used for biological samples, as well as phenotypical classifications. In many cases, retrospective analysis can be used to identify which ion *m*/*z* values contribute the most to the robustness of the model, thereby facilitating the identification of biomarkers. Clinical use of MS has been praised due to a higher fidelity compared to immunoassays.^37,38^ With mass spectral analysis expedited by using ML, we anticipate that the trend of coupling the two methods for clinical diagnosis to continue. Below we will list and describe recent uses of ML to classify MS samples outside the scope of single cell MS (Section 3.4), mass cytometry (Section 3.5), and MSI (Section 3.6).

**Figure 4:**
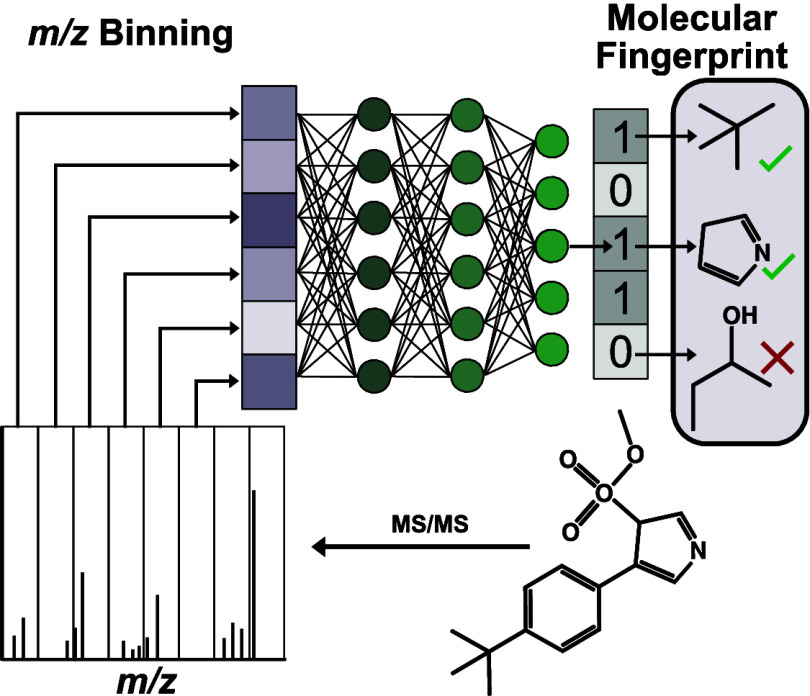
Example schematic for delineating features in molecular structures from mass spectra. Showing a schematic of fully connected feed-forward neural network using binned *m*/*z* values as input and molecular fingerprint as output. Note that binning of *m*/*z* values, though a common practice to provide standardized lengths, results in a loss of information as neighboring ion intensities are grouped into bins.

Random forest models have been used to classify ion-mobility (IM) CID MS/MS data of protonated biomolecules by using custom ontologies, including classifiers like “organic compounds” and “benzenoids”, indicating kingdom and super class.^39^ For the determination of aerosol composition, a pipeline was developed where multinomial logistic regression was used to classify aerosols based on mass spectra, and subsequent fractional composition was predicted using boosted trees on the probabilities generated by the logistic model.^40^ Notably, to combat potential information loss during ion *m*/*z* binning arising from the combination of neighboring *m*/*z* intensities (Figure 4), Imamura and co-workers reported the classification of silane films using a CNN on literal image (.jpg’s not MSI’s) representations of TOF mass spectra.^41^ In an additional example, the Musah group utilized direct analysis in real time (DART) HRMS to identify maggot-derived compounds by employing a combination of hierarchical classification trees and an MLP.^42^ We note that this report contains excellent descriptions of model selection and post training validation.^42^ Fish species have also been classified using ML by using rapid evaporative ionization MS. Here, it was found that PCA followed by linear discriminate analysis yielded higher accuracy than RF or SVM.^43^ In a final example, RF was employed to classify spectra collected from headspace solid-phase micro extractions of leaves using a portable GC-MS procedure, facilitating the detection of Huanglongbing disease in navel oranges.^44^ Based on this approach, samples were effectively classified as either healthy, asymptomatic (infected), and symptomatic (infected) based on measured mass spectra.^44^

Recent work has also applied ML to classify clinical MS data. Notable examples include colorectal liver metastasis diagnosis using linear regression on Probe-ESI mass spectra,^45^ classification of nephrotic syndrome forms from kidney tissue biopsies by using desorption electrospray ionization MS and SVM,^46^ COVID screening using SVM with MALDI Fourier-transform ion cyclotron resonance (FT-ICR) mass spectra of saliva,^47^ narcotic detection in blood samples using MLPs on LC HRMS data-independent acquisition experiments,^48^ and antimicrobial resistance determination by using MALDI-TOF mass spectra with LightGBM and an MLP.^49^ In an additional example, Seddiki et al. developed a transfer learning method with 1-D CNNs to examine various sample types by using a range of instruments, showcasing successful finetuning of task specific data when data is sparce.^50^

### 3.4 Omics and Single Cell Mass Spectrometry

Broadly speaking, omics-based disciplines refer to the various fields of study in which biomolecules are identified or quantified in complex biological samples. Prominent omics-based disciplines include proteomics, metabolomics, genomics, transcriptomics, and lipidomics. MS has emerged as the analytical tool of choice for omics-based disciplines due to its high sensitivity, selectivity, and versatility. In many omics-based approaches, mass spectral libraries and retention time (RT) information obtained from chromatography-based experiments are used to determine sample composition. However, analysis of omics data is often complicated due to data complexity arising from biological sample variability.^51^ Thus, ML has been tremendously popular for tasks regarding metabolomics and proteomics data analysis. Recent reviews addressing developments of supervised ML methods for metabolomics,^52^ ML applications to proteomics data obtained via data-independent acquisition,^53^ and deep learning^54^ have been published. Next, we describe recent studies involving metabolomics, proteomics, and lipidomics utilizing both ML and MS methods, before introducing the use of ML in single cell mass spectrometry (SCMS)^55^ studies.

EnvCNN is a CNN that identifies proteins by comparing isotopic peaks in experimental and database mass spectra of intact proteins.^56^ The software suite DIA-NN facilitates demultiplexing of precursor ions generated during data independent acquisition proteomics studies.^57^ This approach has recently been extended to trapped ion mobility separation (TIMS) TOF-MS mediated studies.^58^ In short, DIA-NN demultiplexes by calculating 73 comparative peak scores that compare experimental and reference mass spectra in addition to other relevant chemometric quantities. DIA-NN then uses these scores with a linear classifier to identify the peaks for further analysis. Next, selected peaks are given q values by using an ensemble of MLPs, assisting in the reduction of false predictions. In additional examples, RT predictions of cross-linked peptides and small molecules have been achieved with transfer learning with Siamese RNNs^59^ and GNNs,^60^ respectively.

Employing 69 supervised ML algorithms, Li et al. compared classification performance for algae-exposed herbicidal agents by using single cell lipidomic data obtained by MALDI-TOF MS.^61^ Out of all of the models, SVM, linear logistic regression, and flexible discriminant analysis were found to be the most reliable. To predict RT values, deepSCP first employs regularized linear regression by utilizing comparative features that described measured and reported libraries of mass spectra. Next, product ion abundances are predicted with deep learning, and peptide-derived mass spectral matching is achieved by using the outputs of the previous models with LightGBM. Specifically, the deep learning architecture first applies a 1-D convolutional layer on peptide sequences, encoding for charge and post-translation modifications, before processing outputs from the convolutional layer with a bidirectional LSTM. Adding similar utility as RT prediction, the collision cross sections (CCS) of metabolite candidate structures suggested by SIRIUS 4 were predicted using an SVM regression model (CCS Predictor 2.0).^62,63^ Classification of cancer cell phenotypes has been performed using an MLP with the abundances of selected metabolomic biomarkers identified via t-SNE and PCA.^64^ Additionally, various CNNs have been tested for pathogen classification based on SCMS data collected via MALDI-TOF.^65^ As per the reported studies, machine learning has been used on omics data for dimension reduction as well as to identify biomarkers. Lastly, DATSIGMA is a machine learning workflow for the analysis and classification of SCMS data.^66^ Beyond preprocessing, DATSIGMA provides users with the ability to visualize high dimensionality SCMS data using UMAP and also helps extract key features using machine learning models such as SVM, RF, and deep ANNs.^66^ However, SCMS is still in its infancy, and much work is needed to establish a comprehensive ML platform given the versatility of MS methods and predict relationships between genes, proteins, lipids, and metabolites in biological pathways for target and biomarker discovery.

### 3.5 Mass Cytometry

Mass cytometry or cytometry by time-of-flight (CyTOF) MS is an improved technique of flow cytometry, significantly increasing throughput compared with traditional flow cytometry. Briefly, CyTOF uses antibodies coupled with heavy metal isotopes to profile more than 50 protein biomarkers simultaneously without peak overlap in the resulting mass spectra. Further, CyTOF is capable of profiling large amounts of cells within a single sample. However, this leads to challenges in analyzing large data sets with high dimensionality. A popular analysis strategy is using manual gating, a process in which two-dimensional plots are utilized to visualize data and identify cell populations, traditionally by hand or via (semi-)automated computational methods. Unfortunately, 2-D plots are not sufficient to represent complex high-dimensional data. Furthermore, manual gating is time-consuming, prone to human error, and ultimately impractical for large-scale studies, limiting throughput. As a result, ML has been used to aid with the dimensionality reduction of large mass-cytometry data sets for cell subset classifications within each sample. There are plenty of unsupervised or partially supervised machine learning based algorithms such as PCA, t-SNE, Isomap, Citrus, and diffusion map, which can be used to project higher dimension data into lower dimensionality plots.^67−70^

In a recent example, t-SNE and Citrus algorithms were exploited to analyze efficacy data of Pembrolizumab in patients with lung cancer obtained via mass cytometry-based experiments.^71^ Importantly, the results were confirmed by manual gating and PCA analysis. Another notable study utilized an elastic net regularization algorithm called immunological Elastic-Net (iEN).^72^ While they demonstrate success, most deep-learning-based ML methods reported to date need substantially larger sample sizes. However, we note that larger patient cohort sizes for immune profiling are impractical in many clinical settings. In turn, iEN models, such as elastic net (EN) models, can be used for cases that include more features than the number of observations. The iEN model performed substantially well in multiple independent studies that generated mass cytometry data on whole blood as well as a simulated large data set. The model’s improved predictions were demonstrated by the iEN model by multiple independent studies.^72^

To standardize and automate gating processes in mass cytometry, DeepCyTOF methods have been developed that incorporate deep learning neural networks. Interestingly, DeepCyTOF can circumvent run-to-run variations that lead to batch effects in CyTOF data sets. Such variations are known to affect manual gating.^73^ Studies have shown that deep learning models such as DGCyTOF^74^ can be utilized to cluster cells into distinct functional subtypes. Further, the method allows detailed assessment of cellular heterogeneity even for new cell types. This method has been claimed to be better than existing dimension-reduction techniques such as UMAP and t-SNE with k-mean clustering, PCA, Gaussian mixture clustering, and factor analysis. A recent study show that an end-to-end trained deep neural network model is capable of accurately diagnosing the latent cytomegalovirus (CMV) in healthy individuals.^75^ End-to-end training facilitates the direct association of raw mass cytometry data with the clinical outcome rather than using cell subsets identified from manual or automated gating, which could lead to significant information loss. Interestingly, biomarkers associated with the latent CMV infection were identified by interpreting this deep learning model, suggesting the use of these models to associate phenotypical outcome with cytometry data.

### 3.6 Mass Spectrometry Imaging

**Figure 5:**
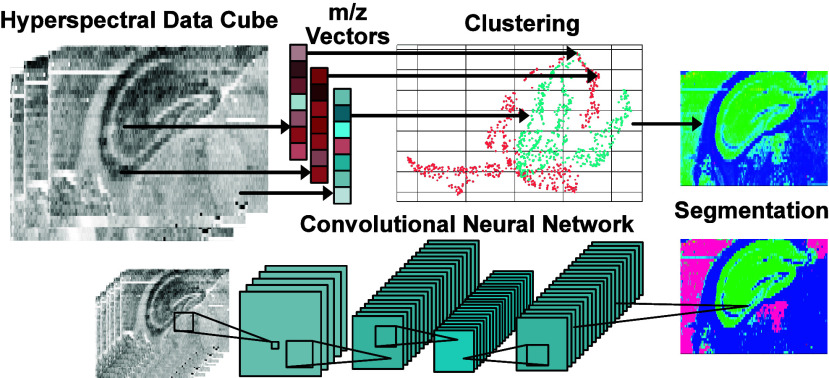
Schematics for mass spectrometry imaging techniques using machine learning for segmentation. Depicting a generalized pipeline for segmenting based on clustering algorithms on ion *m*/*z* vectors of pixels. Segmentation is based on a convolutional neural network.

Mass spectrometry imaging (MSI) records spatial distribution of biomolecules in a label free manner and has been previously reviewed with great detail.^8,76−79^ Similar to images of real-world objects that contain multiple color channels, mass spectral images have multiple channels that correspond to ions with a variety of *m*/*z* values. Generally, several supervised and unsupervised learning approaches can be used to analyze and interpret MSI data (Figure 5). Such approaches include clustering, matrix factorization, manifold learning, and neural networks. Matrix factorization methods like PCA or non-negative matrix factorization methods can be applied for unprocessed MSI spectra to reveal areas of variation. An example of this approach involves using PCA on a stack of MSI spectra measured for coronal rat brain section of a Parkinson’s disease model. The recovered principal components were used to recover brain morphology and show differences between the right and left hemispheres, which is expected in the Parkinson’s disease model.^80^ Clustering methods such as k-means have been used to extract features from the MSI data, where pixels with similar spectral profiles are clustered together. Clusters can then be colored and used to create a single false color image to display additional feature information. Manifold learning can also be used to project spectra from an MSI experiment to 2-D or 3-D visual representations to display regions of variation or interest. Similar to clustering methods, color can be assigned based on the relative positions in 3-dimensional space, which can be used to reconstruct the image, or a single color can be selected to reconstruct a region of interest for the MSI data.^8^

Neural networks have been applied to MSI data using both CNN and RNN architectures. UwU-net has been adapted from U-net, a CNN architecture, and has been modified for high channel MSI data segmentation and classification.^81^ Another recent application of CNNs to MSI data is mi-CNN, which combines multiple instances learning with CNNs for the classification and segmentation of subtissue elements.^82^ Additionally, RNNs have been used to diagnose cancer with MSI data, where an LSTM model was used for capturing the local and nonlocal dependencies in the MSI data and relating them to a cancerous fingerprint, outperformingPCA, LDA, and CNN models.^10^ Lastly, use of a pretrained deep CNN model for embedding was shown to improve downstream spatial clustering of MSI ion images for a UMAP-DBSCAN pipeline, easing with the comparison of entire ion images.^83^ Given the success in computer vision based full CNNs, there are opportunities to use new segmentation methods for MSI to develop single-cell MSI-based omics platforms.

## 4 Future Directions

In recent years, the development of modern ML and deep learning techniques has improved both throughput and fidelity of many computationally driven methods and MS-based approaches. However, there are multiple opportunities yet to be investigated, leaving much to be explored. Additionally, ML and MS synergy standardization practices should be implemented to propel future advancements. In the following section, we discuss envisioned future directions for ML applications in the mass spectrometric field.

### 4.1 Data Standardization and Metadata Usage

Mass spectrometry data display inherent uncertainties associated with experimental factors, such as the ionization method, detector sensitivity, and noise. While human interpretation can typically account for these factors, machine learning methods struggle to address these variations, in part because a large number of small variations in particular input features can dramatically impact overall model prediction.

In order to make generalizable ML models readily applicable to MS-based data, particularly across varying MS platforms, significant efforts are needed toward standardizing mass spectra prior to sharing with scientific community in databases. There are databases that include unprocessed raw mass spectra and chromatograms without *m*/*z* binning or denoising. Additionally, the inclusion of standardized metadata would greatly aid with the usability. By including information such as ionization methods, mass analyzer hardware, and mass resolution, as done in a few databases such as MassIVE, appropriate models can be selected and used without significant modification. Another benefit of including metadata can be its use as a contextual feature for neural networks, such as the tokenization of experimental conditions as inputs for RNNs and transformer networks. The use of contextual features would provide an opportunity for deep learning methods to be generalized and extendable to different experimental setups.

### 4.2 Benchmark Data Sets and Baseline Architectures

With the increasing number of ML model MS-based methods, the need for benchmarking will become ever more crucial. New ML architectures can be highly resource intensive, and many have astutely noted that benchmarking deep learning models against traditional ML models is needed to avoid over engineering.^23^ For meaningful comparisons to be made between ML models, both the data set and evaluation methods need to be uniform. While benchmarking of models and data sets has been reported,^84^ with notable traction in the proteomics^85^ and MSI communities,^86^ the practice has not been uniformly adapted nor standardized across the field. The development of benchmark data sets will be critical for community consensus on the state of the art and the baseline architectures. We hope that the scientific community may find a recently published perspective useful as it discusses the matter in detail useful when developing benchmarking data sets and suites.^87^

### 4.3 Interpretability

The interpretability of the ML models is not uniform across the various models and architectures used for MS-based applications. In some cases, models such as decision trees and linear regression are highly interpretable, whereas deep learning methods are often described as noninterpretable, requiring significant postanalysis interpreation.^54^ However, given the utility of deep learning methods, deep learning architectures should be extended in such a way that results can be easily interpretable or self-interpretable, increasing accessibility to the MS community. This is an active area of research in AI that will have far reaching benefits for the MS community. Additionally, including interpretable measures for model robustness, such as confidence intervals, should help to promote model adoption in practical settings.

### 4.4 Active Learning and Transfer Learning

Incorporating both active learning and domain specific transfer learning toward ML mediated tasks within the mass spectrometry field has the potential to greatly expedite the development and training of new ML models. Active learning is an ML paradigm where the data points for model training are evaluated using a metric such as informativeness, and data points are selected for training in an efficient manner. Though active learning has been used in chemistry, there are not many examples with MS.^88^ Transfer learning is another ML paradigm that also aids in decreasing the required number of labeled data points. Transfer learning is accomplished using pretrained models and fine-tuning them with domain/task specific data. An illustrative example of this would be first training a model to classify animals and then fine-tuning the model by training it to classify less abundant plant photos. By utilizing a larger data set for initially training on a similar “out of domain” task of classifying photos of animals, the model may perform better once fine-tuned for the new “domain” of classifying photos of plants. Transfer learning has already been utilized for MSI,^89^ sample classification,^50,86^ RT prediction,^59^ and spectra refinement.^90^ However, the repurposing and retraining of domain/task-specific models for MS data as starting points remain limited, although some groups have developed domain/task specific transfer learning approaches from the ground up.^89^ The use of MS specific models as starting points for transfer learning will expedite the training process, as well as reinforce the use of MS related baseline models.

## 5 Conclusions

The incorporation of ML into the routine analysis of MS has become increasingly prevalent in recent years, as new approaches continue to be developed across all facets of MS. As novel methods leveraging deep learning and ANNs continue to be applied in the context of MS, it will become imperative to appropriately evaluate and compare ML methods during these exciting times. We hope that this review familiarizes both the MS and ML communities with ML applications to MS-based tasks and expect that the use of ML for MS will continue to rapidly progress by merging these interdisciplinary fields.
